# Supporting Students With Electronic Health Record–Embedded Learning Aids: A Mixed-Methods Study

**DOI:** 10.2196/11351

**Published:** 2019-04-12

**Authors:** Sanne Peters, Geraldine Clarebout, Bert Aertgeerts, Jimmie Leppink, Ann Roex

**Affiliations:** 1 Academic Center for General Practice Department of Public Health and Primary Care Katholieke Universiteit Leuven Leuven Belgium; 2 Center for Instructional Psychology and Technology Department of Psychology and Educational Sciences Katholieke Universiteit Leuven Leuven Belgium; 3 Department of Educational Development and Research School of Health Professions Education Maastricht University Maastricht Netherlands; 4 Hull York Medical School University of York York United Kingdom; 5 Department of Clinical Sciences Faculty of Medicine & Pharmacy Vrije Universiteit Brussel Brussels Belgium

**Keywords:** integrated learning, transfer of learning, electronic health record, electronic performance support system, learning aids, workplace learning

## Abstract

**Background:**

Students often perceive workplace-based learning as disconnected from what they learn in medical school. Interventions that deal with this issue regularly involve feedback and/or learning aids. Feedback has frequently been encouraged in previous research, whereas the use of aids is less understood.

**Objective:**

This study aims to investigate the added value of learning aids in making the connection and enhancing the transfer of learning between medical school and workplace-based learning.

**Methods:**

First-year students in postgraduate general practice training participated in a mixed-methods study. Within a quasi-experimental design, two conditions were investigated: (1) students having access to electronic health record (EHR)–embedded learning aids and feedback and (2) students only receiving feedback. Semistructured interviews were conducted and analyzed according to the thematic analysis approach.

**Results:**

Forty-four students participated in this study. No significant difference was found between the two conditions (*t*_42_=–0.511, *P*=.61, 95% CI –4.86 to 2.90). Nevertheless, students used the aids frequently and found them useful. Given that the aids were familiar to students and contained practice-based instructions in an easily accessible format, they were perceived as feasible to use during workplace-based learning. They also appeared to stimulate transfer of learning, self-confidence, reflection, and interaction between student and supervisor.

**Conclusions:**

Access to EHR-embedded learning aids offers additional support during, but also before and after, patient encounters. The aids can be easily implemented into workplace-based learning.

## Introduction

Education and training programs aspire to the transfer of learning, which is the continuing application of acquired competences in new situations [1-3]. Transfer of learning is important because the goals of training and education are not achieved unless students have the capacity to apply what they have learned in situations that are different from those in which these competences were acquired [4].

Ensuring the transfer of learning has long been recognized as one of the most difficult problems in education [5]. Even though students may do well on an assessment, they will not necessarily do so in a professional context [6]. It has become clear that medical students often encounter difficulties with the transfer of learning when transitioning between the classroom and the clinical workplace [7-10].

One of the principal problems is that students often perceive workplace-based experiences as disconnected from what they learned in classroom sessions (eg, during theoretical sessions, skills training, or simulation-based workshops) [7,8,11]. This lack of connection appears to be often due to two issues. Classroom-acquired competences generally cannot be directly applied in practice [12]. Within medicine, the difficulties in applying information from evidence-based guidelines to individual patients are well documented [13]. Additionally, there is often a delay between learning and the actual application of competences at the workplace and hence the acquired medical knowledge and skills are not so easily retrieved [14]. Consequently, reminders or refreshment of what has been learned before may benefit students.

Resolving these two issues and stimulating the connection between the classroom and clinical workplace has been the focus of many educational interventions [15]. Often these interventions involve a number of learning tools and/or feedback. Learning tools or aids used during classroom learning can support students performing consultations with real patients [14,16,17] to (1) refresh classroom-acquired competences, (2) stimulate deliberate practice at the workplace, and (3) provide just-in-time information during clinical work [15]. Learning aids that reach across both the classroom and workplace might indeed enhance the connection between the two settings and possibly promote transfer of learning [12,18]. Within the medical sector, the electronic health record (EHR) is a platform that can make such learning aids easily accessible across the two settings. Targeting the EHR may offer some benefits. The EHR is already available at the clinical practice and integrated into the workflow [19]. Moreover, the input of registered codes into the EHR generates links to relevant evidence-based information and resources.

Although the EHR plays an increasingly prominent role in health care delivery [20], little is known about offering students access to original classroom-based learning aids through the EHR at the workplace. Hence, in this research, we want to focus on the effect of providing access to EHR-embedded learning aids across both settings. Research shows that the use of learning aids strongly depends on the perceptions students hold about these aids [21]. Even carefully designed learning aids may be used by students in unintended ways or not used at all [22]. Gaining an insight into students’ perceptions about learning aids is important because these influence not only their learning behavior [23], but also the effectiveness of the learning aids and might predict students’ intention for continued use of these aids [21].

Feedback from medical doctors explaining their clinical reasoning can help students to better understand how classroom-acquired competences are translated to decisions in a particular case [24].

Although feedback (and providing support for supervisors’ feedback) has frequently been investigated and encouraged in previous research, the use of classroom-based learning aids is less well understood. This study aims to investigate the added value of learning aids, which are also accessible at the workplace, in making the connection and enhancing the transfer of learning between medical schools and workplace-based contexts. Therefore, the following research questions were examined: (1) Does access to EHR-embedded learning aids in addition to feedback support from supervisors enhance the transfer of learning during medical students’ workplace-based learning experiences? and (2) What are students’ perceptions about using these EHR-embedded learning aids during workplace experiences?

## Methods

### Participants

First-year students in postgraduate general practice (GP) training at the University of Leuven in Belgium were invited to participate in this study on a voluntary basis. The postgraduate GP training is for 2 years and starts after successful completion of the 6-year basic medical course. Students were eligible to participate if they were involved in an internship at a GP clinic between January and May 2016 and performed consultations on patients with acute lower back pain during that period. Recruitment was conducted via an electronic mailing list and face-to-face announcements. At this stage of their training, students participate in an integrated curriculum in which they spend approximately 23 weeks at the university, supplemented by a 6-week internship at a GP and a 14-week internship covering various disciplines at the hospital. The workplace supervisors of the participating students were informed about the study via a telephone conversation, an email, and a letter.

### Study Design and Procedures

**Figure 1:**
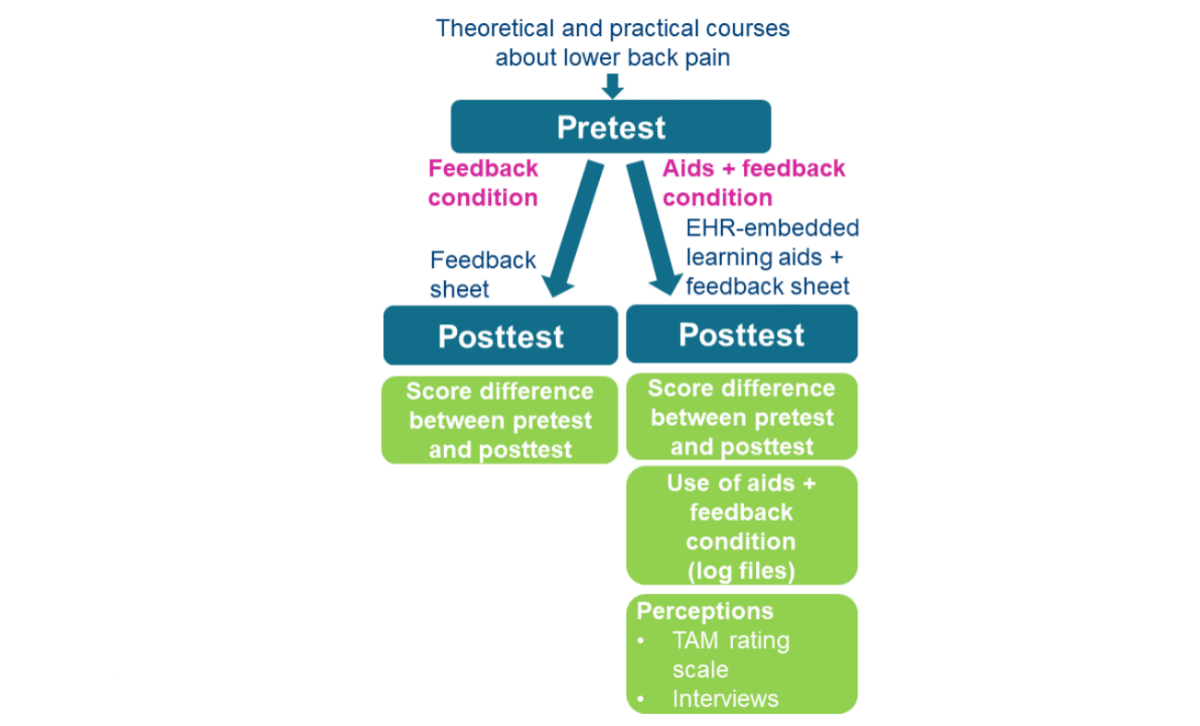
Study design. EHR: electronic health record; TAM: technology acceptance model.

A mixed-methods approach was used, consisting of a quantitative section followed by a qualitative section (see Figure 1) [25]. This study was approved by the Social and Societal Ethics Committee of the University of Leuven (reference number: G-2016 01 437/G-2016 01 438).

#### Quantitative Section

A quasi-experimental design was used. Participants were assigned to one of two conditions depending on the time of their registration for the postgraduate GP course. Random assignment to the conditions was not feasible in this case. One group of students was permitted access to EHR-embedded learning aids and a feedback sheet (aids plus feedback condition), whereas the other group only had access to the feedback sheet (feedback condition). Both groups of students had the same theoretical and practical courses prior to the start of the study. The preintervention test (pretest) took place on the first day following the course, the postintervention test (posttest) after 9 weeks. Students and workplace supervisors were not aware of the two different conditions and, therefore, did not know which one of the two conditions they were assigned.

All workplace supervisors in both the feedback condition and the aids plus feedback condition were asked via a telephone call, before the start of the internship, to allow the students to perform as many consultations as possible with patients suffering from acute lower back pain *.* Additionally, all supervisors of both conditions were asked to provide feedback on the students’ performances. If possible, this feedback followed immediately after the consultation or at the end of the day. Given that the variability of feedback from various supervisors may influence the research findings of the learning aids, it was aimed to standardize the feedback with a feedback sheet, which was provided to all supervisors (see Multimedia Appendix 1) [26]. This feedback sheet contained a definition of feedback and questions that could facilitate the feedback process. It had been revised by three researchers and tested with a potential user.

#### Aids Plus Feedback Condition

**Figure 2:**
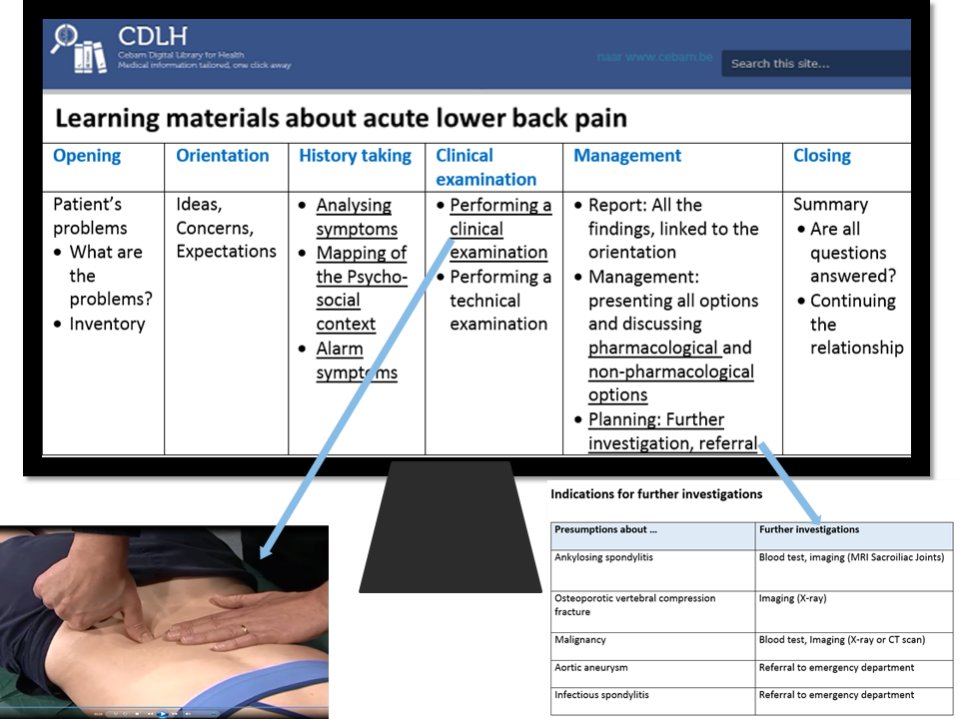
The presented structure of the learning aids.

The students in the aids plus feedback condition were permitted access to EHR-embedded classroom-based learning aids relating to acute lower back pain during their 3-week internship in GP. These learning aids supplemented the existing evidence-based medicine guidelines, already accessible via the EHR. The learning aids were designed by three medical teachers and an educational researcher. They were based on the principles of electronic performance support systems [14,27-29]. The aids consisted of brief, practical, and easily accessible information in various formats (eg, a flowchart, a brief list with procedures, and a short video demonstrating different steps). All the materials were presented according to the sequential phases of a consultation model [30] (see Figure 2), a design with which the students were already familiar. By selecting the hyperlinks, students were able to obtain the corresponding course information. The learning aids were introduced in two initial plenary sessions dealing with theoretical and practical considerations for treating acute lower back pain. Consequently, students were familiar with the aids before the start of their internship. Moreover, the students were trained how to use the EHR and given a demonstration of how to access the EHR-embedded learning aids. During the internship, information could be accessed based on the students’ individual needs and preferences. The aids could be retrieved after registering the potential diagnosis of acute lower back pain in the EHR. Workplace supervisors were also able to access these aids. In contrast to the supervisors in the feedback condition, supervisors in the aids plus feedback condition received an additional letter with a sticky note summarizing their role in the research via postal mail at the beginning of the internship and a reminder via email halfway into the internship due to a holiday period.

#### Feedback Condition

Students in the feedback condition were able to use the EHR in a regular way but were not granted access to the tailored aids via the EHR. Although this student group was permitted access to the learning aids through the course notes, these were not easily accessible at the workplace.

### Outcome Measurements and Instruments

The effect of the EHR-embedded learning aids on the transfer of learning was assessed by the score difference between the performance on a pretest (a few days before the intervention) and a posttest (5 weeks after the intervention). During both tests, students performed one consultation with a standardized patient suffering from acute lower back pain. Each consultation contained three parts: (1) history taking, (2) clinical examination, and (3) management. There were three scenarios for the pretest and three different scenarios for the posttest. Students were randomly allocated to one of these scenarios. Three medical teachers validated the content and the degree of difficulty of all six scenarios. The standardized patients had more than 5 years’ experience performing such roles and were trained to participate in each consultation in a standardized way. The students could not access the EHR-embedded learning aids during the pretest and posttest. In both tests, the consultation took place in a GP practice, and the students’ performances were video recorded. Two observers (JH and JC), who were not aware of the students’ assignment to one of the two conditions, scored the video recordings with a checklist derived from an objective structured clinical examination (OSCE) checklist (see Multimedia Appendix 2). The checklist consisted of 29 items and covered aspects of the whole consultation. The checklist was assessed for face validity and tested by three medical teachers and an educational researcher. The reliability analysis indicated a Cronbach alpha of .84 for the pretest and .72 for the posttest (McDonald omega of 0.84 and 0.75, respectively).

Both observers had many years of experience with scoring OSCEs. All the video recordings were analyzed independently by the two observers and scores were agreed on during a discussion. Moreover, demographic information of the participants and information about the internship (eg, How many acute lower back pain consultations did each perform during the study?) was retrieved via a short questionnaire to the students and the workplace supervisors. Two additional outcome measures were completed by the participants in the aids plus feedback condition. Firstly, the frequency of students’ use of the EHR-embedded learning aids was recorded via log files. Individual student’s use of the aids was linked to their performance on the pretest and posttest. Secondly, students’ perceptions about the usefulness, perceived ease of use, and the intention for continued use of the aids were measured during the posttest via the technology acceptance model (TAM) 6-point rating scale [21,31]. Each item of the TAM rating scale was translated into Dutch and then revised by two researchers using the translation/back-translation method to avoid semantic problems [32].

### Data Analysis

An unpaired *t* test on the difference between groups in pretest to posttest difference was performed using IBM SPSS Statistics for Windows version 24.0 (IBM Corp, Armonk, NY, USA). Additionally, a Bayesian unpaired *t* test was performed because conventional *t* tests (eg, statistical significance tests) may provide some evidence against a null hypothesis (eg, in the case of a statistically significant outcome), but cannot provide evidence in favor of a null hypothesis [33]. The Bayes factor hypothesis testing approach allows for comparing the likelihood of a finding under the null hypothesis with that under an alternative hypothesis [33,34]. The Bayesian analysis was performed in JASP version 0.8.1.1 [35].

### Qualitative Section

Semistructured interviews were conducted with a voluntary sample of the students in the aids plus feedback condition. An attempt was made to gather a wide range of participants, both in terms of their demographics and their experience with the learning aids at the workplace.

The open-ended interview questions were based on three general questions: (1) What are your perceptions about using these EHR-embedded learning aids during clinical practice? (2) Why and how did you use these aids? and (3) What is the feasibility of using such aids at the workplace? The interview questions were revised by three researchers, and two pilot interviews were conducted to test the interview questions and to practice interview techniques. The interviews were recorded and transcribed verbatim. An iterative process of data collection and analysis took place [36]. The principle of data saturation was applied. The interviews were coded and analyzed independently by two researchers (AR and SP), using the software program QSR International’s NVIVO version 11. Following the thematic analysis approach, the initial coding phase focused on small units of the transcripts to ensure that the most prominent ideas were identified. In the second coding phase, broader categories containing a number of conceptually related ideas were developed [37]. Discrepancies in coding were discussed until consensus was reached.

## Results

### Quantitative Section

Twenty-two students participated in the feedback condition. Two of those participants dropped out before the posttest. There were 28 participants in the aids plus feedback condition, of whom four dropped out before the posttest. The data from the participants who dropped out were not taken into account for the data analysis. Descriptive statistics are reported in Table 1.

The checklist to assess students’ performances on the pretest and posttest contained 29 items (Multimedia Appendix 2). Items 28 and 29 were not included in the data analysis because the researchers realized that these two items were too general and difficult to score.

The unpaired *t* test on the difference between groups in pretest to posttest showed that participants in the aids plus feedback condition scored slightly higher than the students in the feedback condition, but this difference was not statistically significant (*t*_42_=–0.511, *P*=.61, 95% CI –4.86 to 2.90). The Bayesian unpaired *t* test revealed a Bayes factor for the null versus the alternative hypothesis of 3.015, indicating some evidence in favor of the null hypothesis of no difference between the two conditions. Without the data of the student in the aids plus feedback condition who did not access the EHR-embedded learning aids, the conventional *t* test was *t*_41_=–0.600 (*P*=.55, 95% CI –5.12 to 2.77) and the Bayes factor for the null versus the alternative hypothesis was 2.877.

The students’ log files in the aids plus feedback condition showed that the EHR-embedded learning aids were used 451 times by students. Students in the feedback condition reported that they did not frequently access the aids through the course notes while on an internship (never: n=13; sometimes: n=5; usually: n=1; always: n=1).

**Table 1:** Descriptive statistics of participants (N=44).

Topic	Feedback condition (n=20)	Aids plus feedback condition (n=24)
Sex (female), n (%)	13 (65)	12 (50)
Age (years), mean (SD)	24.80 (1.44)	24.50 (0.60)
Number of acute lower back pain consultations, mean (SD)^a^	4.47 (3.37)	8.74 (6.22)
Number of feedback received after acute lower back pain consultations, mean (SD)^a^	2.79 (1.84)	4.61 (4.62)
Pretest scores, mean (SD)	17.00 (5.79)	16.13 (5.06)^b^
Posttest scores, mean (SD)	19.35 (4.10)	19.46 (3.72)
Score differences between pretest and posttest, mean (SD)	2.35 (6.79)	3.33 (5.96)

^a^Missing data from one student.

^b^One student did not access the learning aids. Without this student, the scores were pretest mean 15.78 (SD 4.88), posttest mean 19.30 (SD 3.72), and score difference between pretest and posttest mean 3.52 (SD 6.02).

**Table 2:** Students’ perceptions about the learning aids in the aids plus feedback condition (n=24).

Students’ perceptions about the learning aids	Mean (SD)	Cronbach alpha	McDonald omega
Perceived usefulness (4 items)^a^	4.78 (1.04)	.72	0.77
Perceived ease of use (4 items)^b^	4.90 (1.01)	.78	0.81
Intention for continued use (2 items)	4.94 (1.10)	.92	0.92

^a^For one item, there is missing data for one student.

^b^For two items, there is missing data for one student.

The students’ perceptions about the learning aids are shown in Table 2. Students found the aids generally useful, easy to use, and they had the intention to continue using them. Yet, the standard deviation is rather large.

Although the learning aids were developed for the students, 12 of 24 workplace supervisors in the aids plus feedback condition reported that they also used the EHR-embedded learning aids.

### Qualitative Section

Data saturation was reached after 16 interviews with students in the aids plus feedback condition. Table 3 shows a detailed summary of the qualitative findings. The data analysis indicated that all students reported having used the learning aids, but only a small fraction of them had done so during the consultation. The following reasons were given: low confidence or resistance to accessing the EHR during patient encounters, supervisor’s influence (eg, time pressure), patient’s influence (eg, patient’s individual situation), practical limitations, small number of consultations performed with patients suffering acute lower back pain, and in some cases students knew the content of the aids already.

Students who used the learning aids during consultations mentioned that the aids were helpful to check if anything had been forgotten or to clarify uncertainties. Some participants explained that the aids helped them to feel a bit more self-confident. Students described that it was feasible to use the aids during consultations because they were familiar with them as they reflected what was learned before, contained brief and practice-based information with step-by-step instructions, and were presented in an easily accessible format. The beneficial characteristics of the learning aids were perceived to be missing in the general evidence-based guidelines available on the EHR. Although the learning aids were developed for use during consultations, the majority of students used them before and after consultations. Participants mentioned that they used the aids to refresh acquired competences before consultations because they were anxious about making mistakes or perceived weakness in this area of their practice. Some students explained that these aids helped them to be more aware of the learning content and to apply this in the workplace. Students also found it useful to check afterward whether they had performed the consultation correctly. Some students explained that they also used the aids to discuss their performance with their supervisor or to reflect on their supervisor’s performance. The aids were even perceived as tools for supervisors’ professional development.

**Table 3:** Summary of qualitative findings.

Categories and themes		Illustrative quotations
**Reasons for limited or no use during consultations**		
	Low confidence or resistance to accessing (medical evidence through) the EHR (electronic health record) during consultations	“During the history taking, I definitely didn’t go through all alarm signals...because you sometimes have the feeling that you spent too much time on your computer, reading rather than talking to the patient.” (student 3)
	Supervisor’s influence: time pressure, performing part of consultation, asking clinical questions, mimicking habits	“I never decided on the duration of incapacity to work...because often the supervisor decided that.” (student 3)
	Patient’s influence: patient knows back pain, resistance to EHR, patient’s individual situation	“Often it was the patients who were known to have back pain...and knew themselves what was going on...You cannot tell each time the same thing to that patient.” (student 1)
	Practical limitations	“I didn’t use it [aids] much...because I often couldn’t access it due to problems logging into the EHR.” (student 12)
**Reasons for limited or no use in general**		
	Limited consultations; content was known	“I think that if I had more patients with lower back pain, I would have had more opportunities to use it [the aids].” (student 5)
**Reasons for use of learning aids (before, during, and/or after consultations)**		
	To refresh acquired competences	“You can definitely not miss the alarm signals...and then you check that [the aid about alarm signals] an extra time.” (student 13)
	To check	“For me, it also serves as reassurance...because I’ve got a bit of stress about how you need to handle it...just because it is visible on my computer...it gives you something to hold on to...just to have some security...I find it really gives me peace of mind.” (student 3)
	Interested: curiosity, participation in study	“I was unsure of the management plan I proposed there [pretest] but if you check the aids, then you remember again how you should do it. That helped me.” (student 6)
**Characteristics of learning aids that stimulate transfer**		
	Content reflected what was learned before; practice-based information; user-friendly; easily accessible format; just-in-time information	“That you don’t need to look at it for a long time...that you don’t need to fully concentrate...I mean, you just read it and it comes back to you.” (student 4)
**Learned from the whole study design**		
	More independence; Pretest as extra practice moment; feedback sheet stimulated supervisors to give more feedback	“That we had to do that test [pretest], then you have repeated it [learning content] very well for yourself and then you spent much time on it...the fact that it is a test and it will be video recorded and it is with a simulation patient, you really would like to perform well.” (student 7)

## Discussion

### Overview

Students thought it was very useful and feasible to use the EHR-embedded learning aids because they were familiar with them and they contained brief, practice-based, step-by-step instructions presented in an easily accessible format. Moreover, the aids were frequently used by students. This suggests that they addressed a need for this kind of support. Students described that the aids facilitated transfer of learning because they allowed them to refresh and check classroom-acquired competences at the workplace. Additionally, the aids appeared to give students more self-confidence, supported students’ reflections, and stimulated interactions with supervisors. Yet, no significant difference was found between offering support for feedback with the additional access to EHR-embedded learning aids and solely providing support for feedback without the additional aids.

### Comparison With the Literature

Participants in this study described that the learning aids often could not be directly applied in practice because they required tailoring to a patient’s circumstances, as pointed out in previous research [8]. Transfer research emphasizes that transfer is not a simple “store and retrieve process” but involves active interpreting, modifying, and reconstructing the competences to be transferred [12]. To help students better understand how classroom-acquired competences are transformed into clinical decisions for a particular patient, feedback from the supervisor is essential [24,26]. Some participants mentioned that the feedback sheet stimulated their supervisors to give better feedback.

Additionally, students in this study mentioned that their use of the learning aids also depended on their supervisor. This is in line with previous research that shows that evidence-based medicine (EBM) decision-making processes are influenced by patient factors as well as general practitioner factors (eg, time pressure, attitude, and experience) [13]. Also, the GP supervisor’s role as a model for students is important because their attitude toward the use of EBM and learning aids may be a barrier for students [38,39]. This is especially the case for GP students and trainees because they generally have just one supervisor [40].

Students in this study found the way in which the aids reflected previously acquired competences was beneficial for transfer of learning. This is in line with previous research that showed that spaced repetition of what was learned before promotes students’ memory, problem solving, and transfer of learning [41]. Moreover, research indicated that familiarity with learning aids is one of the crucial elements for medical doctors and students to use these aids [19]. Among other reasons, familiar resources might help with finding answers to point-of-care questions more quickly than unfamiliar resources [19,42]. This is especially relevant for students because it allows them to focus on the application of classroom-acquired competences rather than losing time searching for the desired information [43]. Yet, the learning aids in this study may not be up-to-date after a period of time. Nevertheless, the aids were not intended as a replacement of evidence-based guidelines but rather as a supplement.

Participants in this study explained the added value of learning aids alongside evidence-based guidelines. The latter often consist of long passages of text and are perceived to be too complex to be useful, often requiring a lot of time for searching and reading [13]. Some advantages of the learning aids in this study were their brevity, practice-orientation, and step-by-step design in an easily accessible format.

Previous research indicates that practical tools can encourage transfer of learning [8,15]. Moreover, these practical tools or aids can be adjusted to students’ individual needs or extended with students’ personal documents so that they become “personalized learning aids” [29]. This allows richer and deeper personalized learning experiences [22,44]. Additionally, students can become actors to develop classroom-based learning aids themselves, which may be shared with other students [22,28,44,45]. Additionally, students and supervisors could work together on such projects to bring classroom and workplace learning closer together. Previous research indicated that collaboration between supervisors and students can take place by involving students in a shared project [12,42]. Moreover, learning aids could also be based on existing sources available at the workplace, such as evidence-based guidelines. Bringing such sources into the classroom more frequently might allow students to familiarize themselves with them.

The EHR-embedded learning aids have a lot in common with electronic performance support systems. These are electronic systems that offer support during performances at the workplace [29]. They are mainly used during workplace performances but can also be used prior to and after performance [29]. Rather than using the learning aids during patient encounters, as was the intention of the researchers, most participants of this study used the learning aids before and/or after patient encounters. This is in contrast with previous research that showed that students or trainees primarily seek answers to clinical questions during patient consultations [46,47]. Yet, it indicates that students’ individual perceptions influence how they use educational support, as shown by previous research [21,22].

### Limitations

A limitation of this study was the small number of participants and the limited intervention period. Nevertheless, the use of both a quantitative and a qualitative approach in the study design was a strength. This allowed investigation of students’ competences and perceptions as well as more insight into students’ use of the learning aids. Yet, interviews were only conducted with the aids plus feedback condition. Interviews with the feedback condition could have been informative too. Another limitation was that pretests were used although it is known that these are not learning neutral. They may stimulate learning to the test and affect performance on an identical posttest [48], which could have diminished the effect of the learning aids. Another limitation was that the pretest and posttest only contained one OSCE. Yet, it was a strength that the OSCE in this study was based on a whole consultation rather than short stations assessing clinical competences in isolation. The use of whole consultations and integrated competence assessments more closely reflect the real patient encounter [49]. Additionally, given that the OSCEs in this study were videotaped, it allowed the two observers to review elements that they were unsure of on the first viewing [50]. Regrettably, individual student use of the learning aids could not be linked to their performance on the pretest and posttest. Although it was the researchers’ intention to do so, it was impossible due to a failure of the logging system. Another limitation was that there was no information regarding the duration of time in which students accessed the learning aids. This study focused on the use of EHR-embedded learning aids, but “use” does not automatically translate to the application in practice. Previous research indicated that using EBM (or learning aids) may be important, but alone it is insufficient to improve clinical practices or patient care [51]. It was a limitation of the study that the quality of the received feedback was not assessed. Yet, materials and instructions were provided to generalize its format. The study results showed that students in the aids plus feedback condition were able to perform more consultations and they received more feedback than students in the feedback condition, which may have influenced the results. It is possible that the extra reminder via email and postal mail for supervisors in the aids plus feedback condition plays a role, although this remains unclear. It might, for example, also be possible that students took more initiative because they had learning aids available in the workplace. Furthermore, random assignment to the conditions was not feasible in this study. Given that the students ended up in two groups by a mechanism (time of registration for the postgraduate GP course) unrelated to the two conditions, it was assumed to be sufficient. It was a strength of this study that participants were unaware of their assignment to one of the two conditions.

### Practical Implications and Future Research

During patient encounters, there is often not much time to search for information in evidence-based guidelines [13]. This study showed that information that is easily accessible, user-friendly, and familiar to students facilitates the information search. This raises the question whether changing the format of evidence-based guidelines and implementing a design more in line with the characteristics of the learning aids in this study (practice-based, step-by-step instructions and easily accessible format) may be helpful. This study also indicated that students often consulted the learning aids before or after patient encounters, provided they were familiar with the aids. This implies that access should be possible at any time and available aids should be made familiar by using them in classroom-based learning activities. This study also showed that students felt restrained when using learning aids during patient encounters, although they perceived the aids as useful, easy to use, and an added value above the existing evidence-based guidelines. This study provides insight into the interfering factors in the transfer of learning, such as the supervisors’ role model. Future research should explore how these interfering factors could be overcome (eg, the influence of supervisors’ educational training).

### Conclusion

Access to EHR-embedded learning aids, in addition to feedback, did not seem to enhance transfer of learning based on the results of the pretest and posttest. However, students perceived the aids as helpful in their transition from medical school to the workplace. They expressed that the learning aids offered additional support during, but also before and after, patient encounters. The learning aids can be easily implemented into workplace-based learning to assist both students and supervisors.

## Appendix

Multimedia Appendix 1 Feedback sheet and cognitive feedback.

Multimedia Appendix 2 OSCE checklist Acute Lower Back Pain.

## Abbreviations

EBM: evidence-based medicine
EHR: electronic health record
GP: general practice
OSCE: objective structured clinical examination
TAM: technology acceptance model

## References

[1] Barnett SM, Ceci SJ (2002). When and where do we apply what we learn? A taxonomy for far transfer. Psychol Bull.

[2] Blume BD, Ford JK, Baldwin TT, Huang JL (2009). Transfer of training: a meta-analytic review. J Manage.

[3] Cheng E, Hampson I (2008). Transfer of training: a review and new insights. Int J Manage Rev.

[4] Subedi B (2004). Emerging trends of research on transfer of learning. Int Educ J.

[5] Haskell R (2001). Transfer of Learning.

[6] Woodworth Rs, Thorndike El (1901). The influence of improvement in one mental function upon the efficiency of other functions. Psychol Rev.

[7] O'Brien B, Cooke M, Irby DM (2007). Perceptions and attributions of third-year student struggles in clerkships: do students and clerkship directors agree?. Acad Med.

[8] Peters S, Lepeleire J, Cortvriendt S, Keyser C, Hoet K, Janssens B, Roex A (2015). Transferring high quality care of the elderly into the clinical workplace: barriers and facilitating factors. BJMMR.

[9] Prince KJ, Van De Wiel M, Scherpbier AJ, Can Der Vleuten CP, Boshuizen HP (2000). A qualitative analysis of the transition from theory to practice in undergraduate training in a PBL-medical school. Adv Health Sci Educ Theory Pract.

[10] Yardley S, Brosnan C, Richardson J, Hays R (2013). Authentic early experience in medical education: a socio-cultural analysis identifying important variables in learning interactions within workplaces. Adv Health Sci Educ Theory Pract.

[11] Yardley S, Brosnan C, Richardson J (2013). The consequences of authentic early experience for medical students: creation of mētis. Med Educ.

[12] Tuomi-Gröhn T, Engeström Y (2003). Between School and Work: New Perspectives on Transfer and Boundary-Crossing.

[13] Hannes K, Goedhuys J, Aertgeerts B (2012). Obstacles to implementing evidence-based practice in Belgium: a context-specific qualitative evidence synthesis including findings from different health care disciplines. Acta Clin Belg.

[14] Chang C (2007). The relationship between the performance and the perceived benefits of using an electronic performance support system (EPSS). Innov Educ Teach Int.

[15] Peters S, Clarebout G, Diemers A, Delvaux N, Verburgh A, Aertgeerts B, Roex A (2017). Enhancing the connection between the classroom and the clinical workplace: a systematic review. Perspect Med Educ.

[16] Yelon SL, Ford JK, Golden S (2013). Transfer over time: stories about transfer years after training. Perf Improvement Qrtly.

[17] Boehlecke B, Sperber AD, Kowlowitz V, Becker M, Contreras A, McGaghie WC (1996). Smoking history-taking skills: a simple guide to teach medical students. Med Educ.

[18] Konkola R, Tuomi‐Gröhn T, Lambert P, Ludvigsen S (2007). Promoting learning and transfer between school and workplace. J Educ Work.

[19] Cook DA, Sorensen KJ, Hersh W, Berger RA, Wilkinson JM (2013). Features of effective medical knowledge resources to support point of care learning: a focus group study. PLoS One.

[20] Campanella P, Lovato E, Marone C, Fallacara L, Mancuso A, Ricciardi W, Specchia ML (2016). The impact of electronic health records on healthcare quality: a systematic review and meta-analysis. Eur J Public Health.

[21] Venkatesh V, Morris MG, Davis GB, Davis FD (2003). User acceptance of information technology: toward a unified view. MIS Quarterly.

[22] Sandars J (2011). It appeared to be a good idea at the time but … A few steps closer to understanding how technology can enhance teaching and learning in medical education. Med Teach.

[23] Entwistle N (1991). Approaches to learning and perceptions of the learning environment. High Educ.

[24] Pinnock R, Young L, Spence F, Henning M, Hazell W (2015). Can think aloud be used to teach and assess clinical reasoning in graduate medical education?. J Grad Med Educ.

[25] Creswell J (2003). Research Design: Qualitative, Quantitative, and Mixed Methods Approaches.

[26] Peters S, Clarebout G, Van Nuland M, Aertgeerts B, Roex A (2017). How to connect classroom and workplace learning. Clin Teach.

[27] Schaik Pv, Pearson R, Barker P (2002). Designing electronic performance support systems to facilitate learning. Innov Educ Teach Int.

[28] Milheim W (1997). Instructional design issues for electronic performance support systems. Br J Educ Technol.

[29] Rossett A, Gautier-Downes J (1991). A Handbook of Job Aids.

[30] Brems M, Van Nuland M, De Lepeleire J (2012). Het leuvens consultatiemodel: Peilen naar de ideeën, beleving en verwachtingen van de patiënt [The consultation model of Leuven: investigating ideas, experiences and expectations of the patient]. Huisarts Nu.

[31] Davis FD (1993). User acceptance of information technology: system characteristics, user perceptions and behavioral impacts. Int J Man Mach Stud.

[32] Behling O, Law K (2000). Translating Questionnaires and Other Research Instruments: Problems and Solutions.

[33] Rouder JN, Speckman PL, Sun D, Morey RD, Iverson G (2009). Bayesian t tests for accepting and rejecting the null hypothesis. Psychon Bull Rev.

[34] Leppink J, O'Sullivan P, Winston K (2017). Evidence against vs in favour of a null hypothesis. Perspect Med Educ.

[35] (2018). JASP (Version 0).

[36] Watling CJ, Lingard L (2012). Grounded theory in medical education research: AMEE Guide No. 70. Med Teach.

[37] Braun V, Clarke V (2006). Using thematic analysis in psychology. Qualitative Research Psychol.

[38] Te Pas E, van Dijk N, Bartelink MEL, Wieringa-De Waard M (2013). Factors influencing the EBM behaviour of GP trainers: a mixed method study. Med Teach.

[39] Paice E, Heard S, Moss F (2002). How important are role models in making good doctors?. BMJ.

[40] Allan GM, Ma V, Aaron S, Vandermeer B, Manca D, Korownyk C (2012). Residents' clinical questions: how are they answered and are the answers helpful?. Can Fam Physician.

[41] Kang SH (2016). Spaced repetition promotes efficient and effective learning. Pol Ins Behav Brain Sci.

[42] Yelon SL, Ford JK, Anderson WA (2014). Twelve tips for increasing transfer of training from faculty development programs. Med Teach.

[43] Young JQ, Van Merrienboer J, Durning S, Ten Cate O (2014). Cognitive Load Theory: implications for medical education: AMEE Guide No. 86. Med Teach.

[44] Bricon-Souf N, Leroy N, Renard J (2010). Augmented notebooks for pervasive learning in medical practice. Stud Health Technol Inform.

[45] Crabtree EA, Brennan E, Davis A, Squires JE (2017). Connecting education to quality: engaging medical students in the development of evidence-based clinical decision support tools. Acad Med.

[46] Kortekaas MF, Bartelink ME, Boelman L, Hoes AW, de Wit NJ (2015). General practice trainees' information searching strategies for clinical queries encountered in daily practice. Fam Pract.

[47] McCord G, Smucker WD, Selius BA, Hannan S, Davidson E, Schrop SL, Rao V, Albrecht P (2007). Answering questions at the point of care: do residents practice EBM or manage information sources?. Acad Med.

[48] Cook DA, Beckman TJ (2010). Reflections on experimental research in medical education. Adv Health Sci Educ Theory Pract.

[49] van Merriënboer J, van der Vleuten C, Mayrath MC, Clarke-Midura J, Robinson DH, Schraw G (2011). Technology-based assessment in the integrated curriculum. Technology-Based Assessments for 21st Century Skills.

[50] Lucas NC, Walker N, Bullen C (2016). Using a videotaped objective structured clinical examination to assess Knowledge In Smoking cessation amongst medical Students (the K.I.S.S. Study). Med Teach.

[51] Fiander M, McGowan J, Grad R, Pluye P, Hannes K, Labrecque M, Roberts NW, Salzwedel DM, Welch V, Tugwell P (2015). Interventions to increase the use of electronic health information by healthcare practitioners to improve clinical practice and patient outcomes. Cochrane Database Syst Rev.

[52] Wigton RS (2010). Social judgement theory and medical judgement. Think Reasoning.

[53] van Merriënboer J, Kirschner P (2013). Ten Steps to Complex Learning: A Systematic Approach to Four-Component Instructional Design.

