# Supplementary material for: Supporting Students With Electronic Health Record–Embedded Learning Aids: A Mixed-Methods Study
Source: JMIR Med Educ. 2019 Apr 12;5(1):e11351. doi: 10.2196/11351 (PMC6484259; doi:10.2196/11351)
Supplement: Multimedia Appendix 1 [file mededu_v5i1e11351_app1.pdf]

Feedback sheet for supervisors

➔ Give feedback to the student (more information on page 2)

➔ Feedback is important for the student to be able to learn as much as possible during workplace-based experiences

1. **Ask the student:**
   - How do you think the patient encounter went?
   - In which way did it go as you expected?
   - In which way did it NOT go as you expected?
   - Are there parts that you would have liked to do differently?

   Comment: Draw attention to the history taking, clinical examination and management part of the consultation

2. **Explain your own clinical reasoning:**
   - Clarify how you would have done it.

3. **Conclude:**
   - What will each of you take from this experience?

Cognitive Feedback

This feedback sheet can help you to give "cognitive feedback" to your student. This cognitive feedback is important for the student because it will contribute to the improvement of his clinical reasoning process and, therefore, optimise the patient encounter.[1,2]

Cognitive feedback is:	Cognitive feedback is NOT:
• Asking the student to reflect on the quality of his clinical reasoning process<br><br>• Explaining your clinical reasoning to the student	• Correcting the student's mistakes without engaging him and letting him reflect on it

Cognitive feedback allows the student to critically compare pros and cons about applying certain clinical reasoning processes. This is important as the difference between correct and wrong solutions is not always clear in the medical sector.[3,4] Cognitive feedback will help the student to critically reflect and improve patient encounters.

The questions on page 1 can help you to give feedback to students. In an ideal situation, this conversation takes place as quickly as possible after the patient encounter that the students performed.

References

1 Balzer WK, Doherty ME, Oconnor R. Effects of Cognitive Feedback on Performance. Psychological Bulletin. 1989;106(3):410-33.
2 McKendree J. Effective feedback content for tutoring complex skills. Human Computer Interaction. 1990;5:381-413.
3 Wigton RS. Social judgement theory and medical judgement. Thinking and reasoning. 1996;2(2/3).
4 van Merriënboer JJG, Kirschner PA. Ten steps to complex learning: a systematic approach to four-component instructional design. New York: Routledge; 2013.
