# Supplementary material for: Supporting Students With Electronic Health Record–Embedded Learning Aids: A Mixed-Methods Study
Source: JMIR Med Educ. 2019 Apr 12;5(1):e11351. doi: 10.2196/11351 (PMC6484259; doi:10.2196/11351)
Supplement: Multimedia Appendix 2 [file mededu_v5i1e11351_app2.pdf]

OSCE checklist – Acute Lower Back Pain

Student name: …………………………..

Items for all scenarios

	Adequately performed	Not adequately or not performed
**Introduction**		
1. The student asks the patient's reason for his/her visit and lets the patient tell their complaint (using an open-ended question)	O	O
2. The student makes an inventory of all reasons for encounter	O	O
**Orientation**		
3. The student pays attention to ALL aspects of the ICE (Ideas, Concerns and Expectations)	O	O
**History taking**		
The student gains insight into:		
4. Characteristics of the pain (localisation, severity, duration, origin and evolution day/night)	O	O
5. Irradiation of the pain	O	O
6. Influence of rest, movement and posture on complaints	O	O
7. Limitations of activities of daily life (employment situation, …)	O	O
8. Self-care and treatment up until now (which medication + dosage)	O	O
9. Previous episodes of back pain (development + treatment)	O	O
10. Other relevant pathologies	O	O

**Clinical examination**<br><br>11. The student asks the patient to get undressed down to their undergarments (this is necessary to be able to adequately perform item 13, 14 and 17)<br>12. The student evaluates motor function (toe- and heel position + knee- and Achilles reflex)<br>13. The student checks mobility of the spine (bending forward + sideward)<br>14. The student investigates the sensitivity of both legs<br>15. The student performs the Lasègue test<br>16. The student investigates the integrity of both hip joints<br>17. The student palpates the lumbar spine and the Ilio sacral joints in prone position (also percusses the spine for scenario 3, 4 and 6)	O<br>O<br>O<br>O<br>O<br>O<br>O	O<br>O<br>O<br>O<br>O<br>O<br>O
	**Adequately performed**	**Not adequately or not performed**
**Management**<br><br>18. The student acts according to the correct diagnosis or diagnostic hypothesis<br>19. The student links the findings to the ICE of the patient<br>20. The student gives clear information on the level of the patient (e.g. without jargon)<br>21. The student explains correctly the management option(s) to the patient<br>22. The student discusses the management options with the patient<br>23. The student advises correct medication (including correct dosage[1])<br>24. The student gives correct advise about non-pharmacological options<br>25. The student makes correct decisions about referral<br>26. The student talks about the potential incapacity to work	O<br>O<br>O<br>O<br>O	O<br>O<br>O<br>O<br>O

[1] In case the student prescribes something (e.g. vitamins) while this is not necessary, then this item will be scored as "not adequately performed".

(based on profession)<br>27. The student gives correct information on follow-up[2]	O<br><br>O<br><br>O<br>O<br><br>O	O<br><br>O<br><br>O<br>O<br><br>O
**Overall aspects of the whole consultation**<br><br>28. The consultation was structured<br>29. Medical knowledge and skills (e.g. communication) were smoothly integrated	 <br>O<br>O	 <br>O<br>O

Feedback to the student:

- Describe two positive aspects of the patient encounter: …………………………………………………………
  ……
  ……
  ……

- Describe two aspects of the patient encounter that could be improved: …………………………….
  ……
  ……
  ……

[2] The student also explains the expected development and tells when the patient needs to come back (regarding alarm signals), if necessary.
